# Underweight and Weight Change Increases End-Stage Renal Disease Risk in Patients with Diabetes: A Nationwide Population-Based Cohort Study

**DOI:** 10.3390/nu14010154

**Published:** 2021-12-29

**Authors:** Eun Hui Bae, Tae Ryom Oh, Sang Heon Suh, Eun Mi Yang, Hong Sang Choi, Chang Seong Kim, Seong Kwon Ma, Bongseong Kim, Kyung-Do Han, Soo Wan Kim

**Affiliations:** 1 Department of Internal Medicine, Chonnam National University Medical School, 42 Jebongro, Gwangju 61469, Korea; baedak76@gmail.com (E.H.B.); tryeomoh@hanmail.net (T.R.O.); medssh1984@gmail.com (S.H.S.); nuts99@naver.com (E.M.Y.); hongsang38@hanmail.net (H.S.C.); laminion98@gmail.com (C.S.K.); drmsk@hanmail.net (S.K.M.); 2 Department of Internal Medicine, Chonnam National University Hospital, 42 Jebongro, Gwangju 61469, Korea; 3 Department of Statistics and Actuarial Science, Soongsil University, 369 Sangdo-ro, Dongjak-gu, Seoul 06978, Korea; qhdtjd12@gmail.com

**Keywords:** weight change, end-stage renal disease, diabetes, body mass index, waist circumference, national health programs

## Abstract

Weight variability has known as a risk factor for cardiovascular events and mortality. However, its effect on end-stage renal disease (ESRD) development remains controversial. We investigated the relationship between weight change and ESRD risk. Overall, 97,029 patients with DM aged >20 years were selected from the Korean National Health Screening Program 2009–2012. Weight change was defined as differences in body weight from the index year to 2 years later. Newly diagnosed ESRD was observed until 2017 end. Over a 5.1-year median follow-up period, ESRD was newly diagnosed in 7932 (4.81%) DM patients. BMI < 18.5 kg/m^2^ and waist circumferences <85/80 and >100/95 cm were ESRD risk factors. ESRD risk increased with increasing weight change; ≥10% weight loss (hazard ratio [HR], 1.247) followed by ≥10% weight gain (1.247) was associated with a higher HR than ≤5% weight change after adjusting for several confounding factors. The association between weight change and ESRD risk in a subgroup analysis was significantly stronger in patients aged <65 years, without proteinuria, with BMI ≥ 25, with DM duration <5 years, and prescribed less than 3 classes of DM medication. Underweight patients showed higher ESRD risks than overweight patients. Weight loss >10% was associated with the fastest decline in renal function.

## 1. Introduction

Despite the observational studies that reported positive associations between obesity and chronic kidney disease (CKD) or ESRD, there is not much evidence to prove that obesity is an independent risk factor for end-stage renal disease (ESRD) [1,2,3,4,5]. In addition, some studies have shown that obesity does not increase the risk of ESRD in patients with moderate to advanced CKD [6]. Therefore, the association of obesity with ESRD development remains unclear.

The body weight fluctuation has been associated with increased cardiovascular events and mortality in the general population and patients with CKD as well as patients with DM [7,8,9]. A recent national cohort study found that weight change was associated with increased risks of myocardial infarction (MI), stroke, and death from any cause in people with type 2 DM [10,11], but the effect of weight change on ESRD has not yet been studied in patients with DM.

Therefore, in this study, the effect of body mass index (BMI) and waist circumference (WC) on ESRD development was investigated. In addition, we assessed the relationship between weight change and risk of ESRD in patients with type 2 DM and whether this relationship was affected by baseline BMI, weight change, or stage of advanced diabetes.

## 2. Materials and Methods

### 2.1. Korean National Health Insurance Service Data

This study used the database of the Korean National Health Insurance Service (NHIS), which includes all data claims and the Medical Aid programs. The Korean NHIS database is representative of the entire population of the Republic of Korea, and the details of this database have been described previously. [12]. All insured persons receive medical examinations supported by the National Health Insurance Corporation every year or every other year, depending on their occupation. The database includes sociodemographic data and all medical expenses for inpatient and outpatient services, pharmacy dispensing claims and death information. Anonymous data is disclosed in the National Health Insurance sharing service. This study was approved by the Chonnam National University Hospital Institutional Review Committee (CNUH-EXP-2021-321), and was conducted in accordance with the Human Experiment Committee’s ethical standards and the Declaration of Helsinki. Revised in 1975 and 2013.

### 2.2. Patients

**Figure 1:**
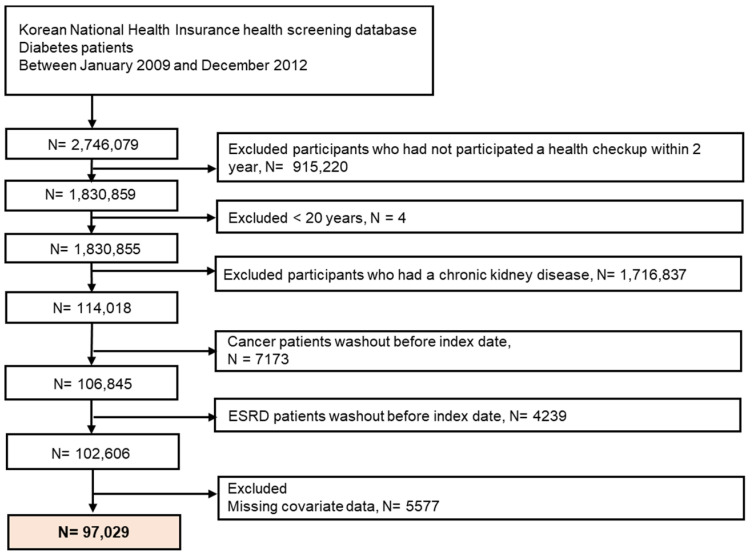
Flowchart of participant enrolment.

Initially, 2,746,079 patients with diabetes who underwent health check-ups from 2009 to 2012 were identified. Of these, we included patients with diabetes who underwent a repeat health check-up after 2 years. The index date was the date of the last health check-up. We excluded those aged <20 years because ESRD development is rare in this subpopulation, having malignancy or with a history of ESRD prior to the index date, and those with missing health examination data. Subsequently, 97,029 patients with DM were included in this study. Figure 1 shows a detailed flowchart of the patient selection. The participants were followed up until ESRD was newly diagnosed or censored by death, loss of health insurance qualification, or end of study (31 December 2019).

### 2.3. Definitions

A DM patient was defined as follows: (i) Insurance with at least one claim per year for the prescription of antidiabetic drugs under International Statistical Classification of Diseases (Tenth Revision, ICD-10) Codes E11–14 claim data, or (ii) fasting blood glucose ≥126 mg/dL at medical examination without prescription for diabetes medication [13,14,15]. Antidiabetic drugs include sulfonylureas, metformin, DPP4 inhibitors, thiazolidinediones, alpha-glucosidase inhibitors, meglitinide, and insulin. Patients with type 1 diabetes (ICD-10 code E10) were excluded from this study. ICD-10 diagnostic code was defined as a comorbidity as in previous studies along with medical use and drug or health checkup results [16,17,18].

Bodyweight change was calculated as over 2 years interval according to the difference in weight values between the first and second health check-up, expressed as a percentage. Based on the previous study [19], we defined the weight stable group as weight change within 5%, and we divided the individuals into five subgroups as follows: ≥10% weight loss, 5–10% weight loss, <5% weight change, 5–10% weight gain, and ≥10% weight gain. We did obtain the intentional weight loss or gain of patients in this study. 

Hypertension was defined as a previously diagnosed hypertension according to ICD-10-codes (I10–I13, I15) or a recorded systolic blood pressure of 140 mmHg or higher (regardless of diastolic blood pressure) or diastolic blood pressure ≥90 mmHg [16,17,18]. Serum creatinine was measured using the traceable Jaffe method, and CKD was defined as an estimated glomerular filtration rate (eGFR) <60 mL/min/1.73 m^2^ and was calculated using the Modification of Diet in Renal Disease formula [20]. Dyslipidemia was defined as a presence of ICD-10-CM code E78, and a history of lipid-lowering drug use or a total serum cholesterol concentration of ≥240 mg/dl in the health examination data. A low income is defined at the lowest 20% of socio-economic status. Body mass index (BMI) was calculated as the weight (in kilograms) divided by the height (in meters squared) according to World Health Organization recommendations for Asians. Smoking history was categorized as never, former, or current smoker. Alcohol consumption was categorized into none, moderate, or heavy drinkers (≥30 g of alcohol per day). Regular exercise was defined as moderate physical activity for at least 20 min per day and >5 days during the week. Participants were tested for proteinuria using the dipstick method. Proteinuria status was defined as negative or positive (dipstick method 1+ or higher for both primary and secondary health examinations).

### 2.4. Outcomes

The study endpoint was an event ESRD defined using a combination of ICD-10 codes (N18-19, Z49, Z94.0 and Z99.2) and special codes assigned at initiation (V codes). Renal replacement therapy during hospitalization (hemodialysis [HD], V001; peritoneal dialysis [PD], V003) and/or kidney transplantation (KT, V005). All medical expenses for dialysis are refunded using the Health Insurance Review and Assessment Service database. These patients are also enrolled as recipients of special medical assistance. Therefore, we were able to identify all ESRD patients in the entire Korean population and analyze data for all ESRD patients who started dialysis. Codes for treatment or medical billing include V005 for KT, V001 for HD, and V003 for PD. Individuals without prior CKD with a transplant or dialysis cord on the same date as the acute renal failure code were excluded. Patients receiving ongoing renal replacement therapy or acute peritoneal dialysis were also excluded.

### 2.5. Statistical Analyses

Data are presented as the mean ± SD for continuous variables and numbers with proportions for categorical variables. Non-normally distributed variables are presented as geometric means (95% confidence interval [CI]). Inter-group differences were tested using a chi-square test or analysis of variance, as appropriate. The incidence rates of ESRD are presented per 1000 person-years. Multivariable Cox proportional hazard regression analysis was used to estimate the hazard ratios (HRs) and 95% CIs of the risk of fracture associated with the weight changes along with adjustment for age, sex, smoking, alcohol consumption, regular exercise, low-income status, insulin use, number of oral hypoglycemic agents, duration of diabetes, previous history of hypertension and dyslipidemia, and previous bodyweight. We also performed analyses to identify the risk of ESRD according to the weight change over 4 and 6 years between the health check-ups. Subgroup analyses were performed in patients with DM patients according to age (<65 and ≥65-years groups), sex, history of hypertension, duration of diabetes (<5 and ≥5-year groups), presence of proteinuria, BMI (<25 and ≥25 kg/m^2^), use of insulin, and the number of oral hypoglycemic agents (≥3) used. Interaction terms were added to test for effect modification across subgroups. All data analyses were conducted using SAS software (version 9.4; SAS Institute, Cary, NC, USA), and *p* < 0.05 was considered statistically significant.

## 3. Results

### 3.1. Baseline Characteristics According to the BMI and Weight Change Status

The mean duration between examinations was 722.30 ± 188.49 days (median 126 (Q1–Q3, 658–785) days. We defined the second health check-up of two BMI and weight measurements as the baseline characteristics of patients for the development of ESRD. The baseline characteristics of the study population were described by BMI changes (Table 1) and body weight changes (Table 2). The mean age of total patients was 68.9 ± 9.1 years, and 47.4% were men, with a mean eGFR of 46.4 ± 12.7 mL/min/1.73 m^2^ and a mean BMI of 25.1 ± 3.3 kg/m^2^. In this study, the distribution of the population according to BMI change was BMI < 25/<25 (43%), BMI < 25/≥25 (6%), BMI ≥ 25/<25 (8%), and BMI ≥ 25/≥25 (43%); body weight change in the <5% stable weight change group over the 2-year period was 68.5%, while ≥10% weight loss was 5.1%, 5–10% weight loss was 14.2%, 5–10% weight gain was 8.8%, and ≥10% weight gain was 3.4%. The participants with stable weight were younger men, tended to be smokers, consumed alcohol, regularly exercised, had a lower income, and had a higher baseline eGFR compared with other weight loss or gain groups. However, the prevalence of hypertension and dyslipidemia, WCs, and total cholesterol and triglyceride levels increased with increased weight gain.

### 3.2. Association of BMI and WC with the Risk of ESRD

**Figure 2:**
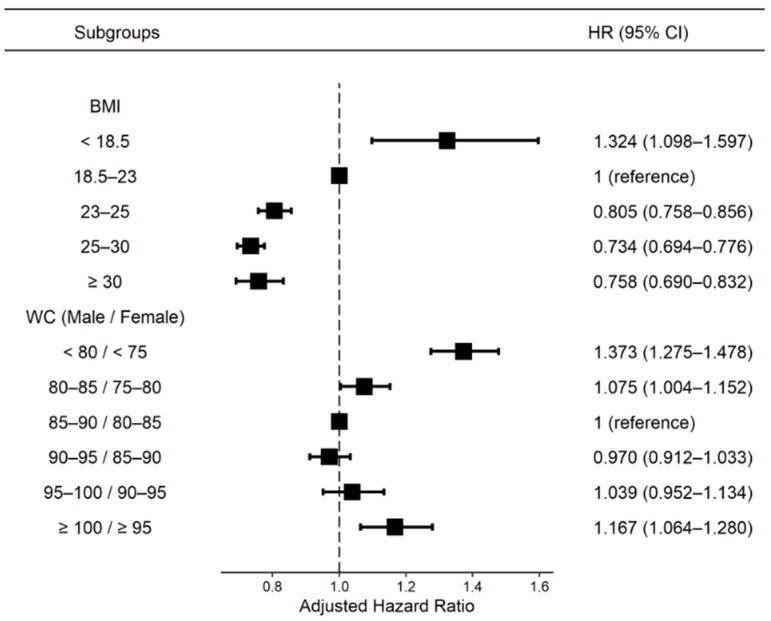
Hazard ratios of ESRD according to the baseline BMI and waist circumference. Adjusted for age and sex, smoking, alcohol consumption, regular exercise, low-income status, use of insulin, number of oral hypoglycemic agents, diabetes duration, and previous histories of hypertension and dyslipidemia. BMI, body mass index; WC, waist circumference; HR, hazard ratio; CI, confidential interval.

Figure 2 shows the risk of ESRD according to BMI or WC. The underweight group with BMI < 18.5 kg/m^2^ (HR: 1.324, 95% CI: 1.098–1.597), the WC < 85/80 cm group (HR: 1.373, 95% CI: 1.275–1.478), and the WC > 100/95 cm group (HR: 1.167, 95% CI: 1.064–1.280) showed higher ESRD risk compared with the reference group (BMI: 18.5–23, WC: 85–90/80–85), while the BMI 25–30 kg/m^2^ group showed the lowest ESRD risk (HR: 0.734, 95% CI: 0.694–0.776) in all participants after adjusting for age, sex, income, presence of hypertension, dyslipidemia, smoking, alcohol drinking, physical activity, glomerular filtration rate, DM duration, use of insulin and number of oral hypoglycemic agents. We also analyzed the results of censoring patients who developed cancer during follow-up to exclude weight loss due to cancer, and the effect of ESRD on weight change was similar to that before cancer censorship (Supplementary Table S1).

### 3.3. Association between BMI or Body Weight Changes and the Risk of ESRD

**Figure 3:**
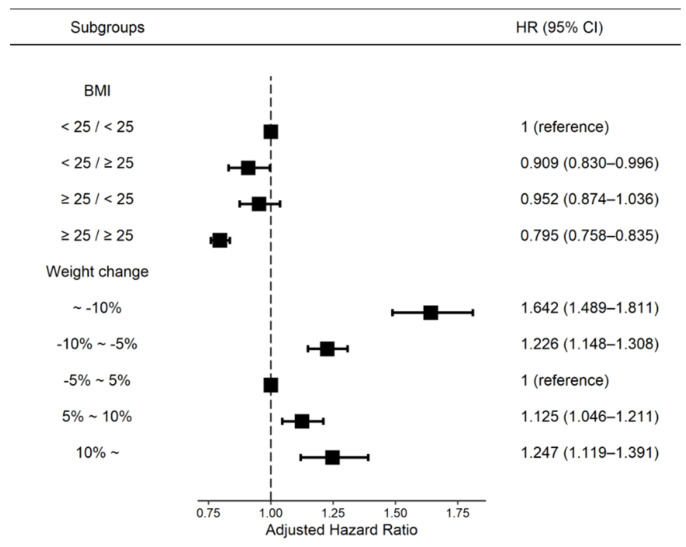
Incidence rates and hazard ratios of ESRD according to the BMI and weight changes. Adjusted for age and sex, smoking, alcohol consumption, regular exercise, low-income status, use of insulin, number of oral hypoglycemic agents, diabetes duration, and previous histories of hypertension, dyslipidemia, and baseline body weight. BMI, body mass index; WC, waist circumference; HR, hazard ratio; CI, confidential interval.

The median follow-up duration was 5.1 years, and incident ESRD was identified in 7932 (4.81%) of the participants over the observation period. The association between BMI or body weight change and the incidence and risk of ESRD in patients with DM is presented in Figure 3. According to BMI change analysis, the BMI ≥ 25/≥25 kg/m^2^ group showed the lowest incident ESRD risk in patients with DM (HR: 0.795, 95% CI: 0.758–0.835) compared to the reference (BMI < 25/<25 kg/m^2^) group. According to the body weight change analysis, patients with ≥10% weight loss over 2 years showed the highest risk for ESRD (HR: 1.642, 95% CI: 1.489–1.811) compared with the reference group (bodyweight change <5%) after adjusting for all covariates (Figure 3, Supplementary Table S2). 

### 3.4. Sensitivity Analyses

Sensitivity analyses were performed to confirm the association between weight change over 4 years and ESRD risk. The adjusted HRs for incident ESRD were 1.756 (95% CI: 1.560–1.976) and 1.255 (95% CI: 1.147–1.374) in patients with ≥10% weight loss and 5% to 10% weight loss, respectively. The risk of ESRD also increased with weight gain ≥10% (HR: 1.338; 95% CI: 1.161–1.542) after full adjustment (Table 3). 

### 3.5. Subgroup Analyses 

**Figure 4:**
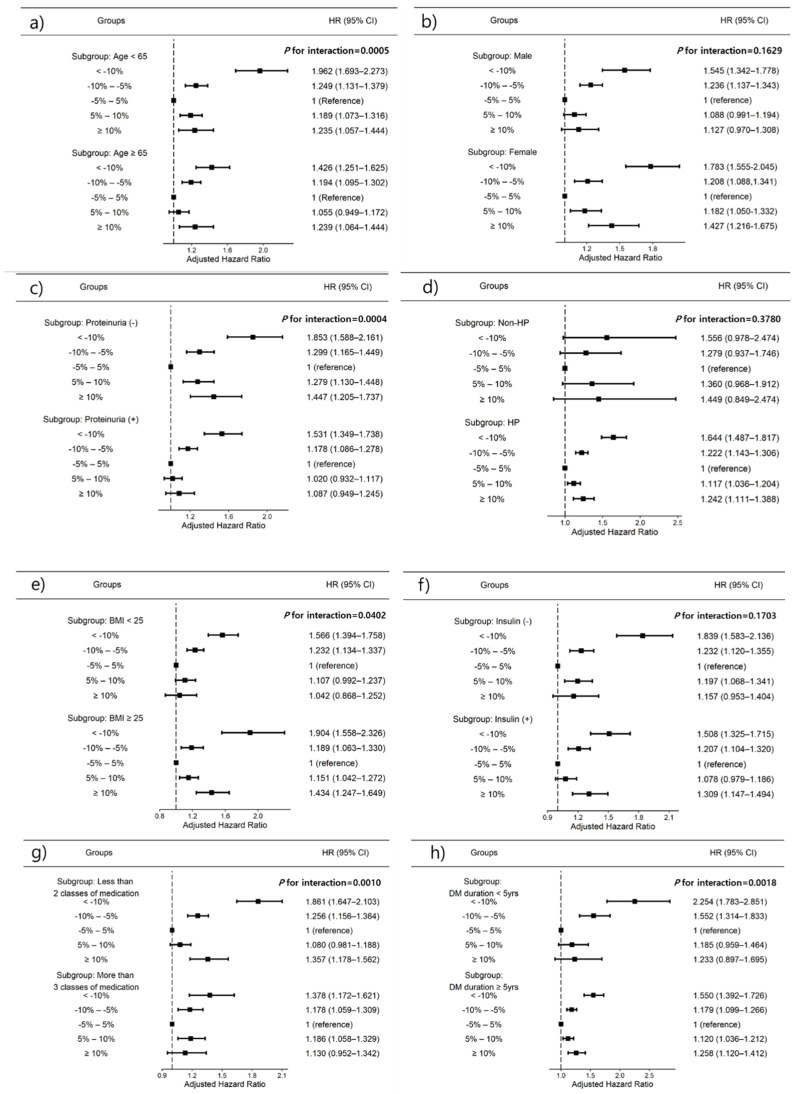
Subgroup analysis for adjusted hazard ratios (HRs) of ESRD. (**a**) Age subgroup, (**b**) sex subgroup, (**c**) history of proteinuria subgroup, (**d**) hypertension subgroup, (**e**) body mass index subgroup, (**f**) use of insulin subgroup, (**g**) use of more than three oral hypoglycemic agent subgroups, (**h**) >5 years DM duration subgroup. Horizontal lines represent the range for 95% confidence intervals (CIs). Models were adjusted for age, sex, smoking, alcohol consumption, regular exercise, income status, insulin use, more than three classes of oral hypoglycemic agents, diabetes duration, previous histories of hypertension and dyslipidemia, and previous body weight. HP, hypertension; BMI, body mass index; DM, diabetes mellitus.

We further explored the association between weight change and risk of ESRD after stratification by age, sex, presence of proteinuria, history of hypertension, BMI, number of oral hypoglycemic agents, insulin use, and DM duration using subgroup analyses (Figure 4). In all subgroup analyses except proteinuria, BMI < 25 kg/m^2^ and more than three classes of hypoglycemic agent use, high weight loss (<10%), or gain (≥10%) were consistently associated with incident ESRD development. The association between weight change and ESRD risk was significantly stronger in patients aged <65 years (*p* for interaction = 0.0005), no proteinuria (*p* for interaction = 0.0004), BMI ≥ 25 kg/m^2^ (*p* for interaction = 0.0402), less than two classes of hypoglycemic agents (*p* for interaction = 0.0010), and those with a diabetes duration of <5 years (*p* for interaction = 0.0018). The weight loss ≥10% group had the highest HRs for all subgroup patients (Figure 4).

## 4. Discussion

The principal findings of the current study can be summarized as follows: (1) underweight (BMI, <18.5 kg/m^2^) but not overweight was a risk factor for ESRD in patients with DM; (2) weight changes of <5% showed the lowest risk for ESRD; (3) weight loss of ≥10% showed the highest risk for incident ESRD; (4) this association was greater in magnitude in patients aged <65 years, with no proteinuria, BMI of ≥25, less than three classes of hypoglycemic agents, and those with a diabetes duration of <5 years; (5) the association between weight changes and ESRD risk was consistent in subgroup analysis. 

The mechanism of obesity-related kidney damage has been explained by hemodynamic changes in the kidney, namely glomerular hyperfiltration, which is augmented by an increased extracellular volume and sodium reabsorption in the proximal tubule and altered tubuloglomerular feedback [21,22,23]. Over time, persistent obesity-induced hyperfiltration leads to thickening of the glomerular basement membrane and contributes to mesangial sclerosis, ultimately leading to renal failure [24,25]. Therefore, the impact of obesity on kidney outcomes has been extensively studied in previous reports [4,26].

Several studies have investigated the association between BMI and the future risk of ESRD. Although conflicting results have been found, most epidemiologic studies have shown that a higher BMI is associated with an increased risk of kidney disease. Two large epidemiologic studies in the United States reported a positive association between BMI and ESRD, and these studies analyzed a broad spectrum of BMI among a large, diverse sample of participants with long-term follow-up for ESRD [4,5]. A higher BMI is estimated to be an independent risk factor for ESRD in any ethnic group. However, the association between BMI and future risk for ESRD tends to be discordant in patients with renal impairment, and this population thus exhibits a so-called “obesity paradox”. In particular, although high BMI is associated with all-cause mortality and decreased renal function in patients with earlier stages of CKD, this association is attenuated in patients with advanced CKD [27,28]. Therefore, controversies remain between BMI and the risk of future ESRD in patients with DM. In this study, unlike the traditional concept of a higher BMI causing metabolic disorders in the general population, an inverse association was found between BMI and the risk of ESRD in patients with DM. This result is similar to findings of previous reports that even among those with obesity or morbid obesity, a higher BMI is associated with better survival in patients with ESRD [27,29]. 

In this study, we investigated the clinical significance of BMI change or body weight change, rather than baseline BMI levels, in patients with DM. In this large-scale cohort, we identified that maintaining a lower BMI (<25/25 kg/m^2^) or >10% weight loss showed the highest risk for ESRD. This significance remained even after the regression model was adjusted for various demographics, lifestyle factors, metabolic parameters, and laboratory parameters. These findings encourage health care providers in the field of nephrology to carefully assess not only baseline BMI values but also BMI changes or weight changes. 

Although the mechanism supporting the association between weight loss and ESRD risk is unclear, changes in body composition, such as muscle mass loss, particularly among the elderly, have been considered [30]. Weight loss is associated with oxidative DNA damage, which is hypothesized to exert a nephrotoxic effect [31] and may also weaken the immune system [32], increasing the incidence of ESRD. 

This study has several limitations. First, we did not collect relevant information on food habits or other comorbidities that might affect weight. Second, this study did not consider the use of medications that affect CKD progressions, such as antihypertensive agents or lipid-lowering agents, and treatment adherence. Third, we were unable to obtain more information about the causes of ESRD. Fourth, we used data from the NHIS checkup program in a Korean population; therefore, we cannot generalize the results to other ethnic groups. Fifth, although we monitored the patients for 5.4 years, the time of follow-up was insufficient for patients to develop ESRD. Lastly, because only body weight was measured, we did not obtain the intentional weight loss or gain of patients in this study, and it was possible that voluntary diet patients for health reasons were included.

To the best of our knowledge, the strengths of the present study were that it is the first study to report the association between body weight change and the risk of ESRD in patients with DM using a large population-based study. Moreover, we found that both weight loss and gain were associated with an increased risk of ESRD in a large sample size of 97,000 Koreans.

## 5. Conclusions

In conclusion, underweight showed a higher risk of ESRD development than those patients with DM who were overweight. Moreover, a weight loss of >10% was associated with the fastest decline in renal function in patients with DM. Therefore, it is important that there are <5% body weight changes to prevent ESRD development in patients with DM. 

## Figures and Tables

**Table 1:** Baseline characteristics of patients according to body mass index change.

Variables	Body Mass Index Change					
	Total (*n* = 97,029)	<25/<25 (*n* = 41,901)	<25/≥25 (*n* = 5851)	≥25/<25 (*n* = 7898)	≥25/≥25 (*n* = 41,379)	*p*-Value
Age, years	68.86 ± 9.11	69.37 ± 9.34	69.15 ± 8.86	70.1 ± 8.77	68.06 ± 8.91	<0.0001
Sex, male	45,955 (47.36)	20,835 (49.72)	2750 (47)	3524 (44.62)	18,846 (45.54)	<0.0001
Smoking						<0.0001
Never	67,046 (69.1)	28,144 (67.17)	4114 (70.31)	5620 (71.16)	29,168 (70.49)	
Former	18,625 (19.2)	8089 (19.31)	1100 (18.8)	1410 (17.85)	8026 (19.4)	
Current	11,358 (11.71)	5668 (13.53)	637 (10.89)	868 (10.99)	4185 (10.11)	
Alcohol consumption						<0.0001
None	75,960 (78.29)	32,821 (78.33)	4542 (77.63)	6430 (81.41)	32,167 (77.74)	
Moderate	18,026 (18.58)	7793 (18.6)	1109 (18.95)	1264 (16)	7860 (19)	
Heavy *	3043 (3.14)	1287 (3.07)	200 (3.42)	204 (2.58)	1352 (3.27)	
Regular exercise	19,929 (20.54)	8939 (21.33)	1173 (20.05)	1547 (19.59)	8270 (19.99)	<0.0001
Hypertension	82,599 (85.13)	33,830 (80.74)	5117 (87.46)	6767 (85.68)	36,885 (89.14)	<0.0001
Dyslipidemia	58,855 (60.66)	23,690 (56.54)	3713 (63.46)	4681 (59.27)	26,771 (64.7)	<0.0001
Income, low 20%	16,657 (17.17)	7153 (17.07)	1047 (17.89)	1324 (16.76)	7133 (17.24)	0.321
eGFR	46.39 ± 12.72	45.67 ± 13.39	46 ± 12.31	46.52 ± 12.37	47.15 ± 12.08	<0.0001
BMI, kg/m^2^	25.06 ± 3.3	22.34 ± 1.75	25.94 ± 1.14	23.87 ± 1.13	27.91 ± 2.43	<0.0001
WC, cm	86.33 ± 8.67	80.78 ± 6.66	88.04 ± 5.98	84.7 ± 6.22	92.01 ± 7.37	<0.0001
SBP, mmHg	130.7 ± 16.4	129.4 ± 16.6	132.2 ± 16.6	129.5 ± 16.4	132.0 ± 16.1	<0.0001
DBP, mmHg	76.9 ± 10.3	75.8 ± 10.3	77.4 ± 10.2	76.4 ± 10.3	77.9 ± 10.2	<0.0001
Tcholesterol, mg/dL	181.0 ± 44.9	179.7 ± 43.7	181.8 ± 44.2	180.0 ± 42.6	182.2 ± 46.5	<0.0001
Triglyceride, mg/dL	142.0 (141.5–142.4)	132.6 (132.0–133.3)	147.7 (145.8–149.6)	137.6 (136.1–39.1)	152.1 (151.4–152.9)	<0.0001
HDL, mg/dL	47.5 ± 14.0	48.2 ± 14.9	47.0 ± 12.6	47.6 ± 16.7	46.9 ± 12.6	<0.0001
LDL, mg/dL	101.6 ± 45.2	101.8 ± 45.7	101.9 ± 46.2	101.8 ± 45.7	101.4 ± 44.6	0.5314
Glucose, mg/dL	131.1 ± 47.9	130.7 ± 49.7	131.8 ± 46.2	130.6 ± 52.8	131.4 ± 45.3	0.1284
Insulin use	25297 (26.07)	11,185 (26.69)	1809 (30.92)	2059 (26.07)	10,244 (24.76)	<0.0001
Anti-diabetes medication						
Sulfonylurea	61,710 (63.6)	26,308 (62.79)	3829 (65.44)	5088 (64.42)	26,485 (64.01)	<0.0001
Metformin	67,768 (69.84)	29,142 (69.55)	4057 (69.34)	5671 (71.8)	28,898 (69.84)	0.0008
Meglitinides	5133 (5.29)	2419 (5.77)	393 (6.72)	368 (4.66)	1953 (4.72)	<0.0001
Thiazolidinedione	9978 (10.28)	3634 (8.67)	791 (13.52)	747 (9.46)	4806 (11.61)	<0.0001
DPP-4 inhibitor	24,295 (25.04)	10,595 (25.29)	1454 (24.85)	2067 (26.17)	10,179 (24.6)	0.0111
α-glu inhibitor	22,237 (22.92)	10,609 (25.32)	1442 (24.65)	1872 (23.7)	8314 (20.09)	<0.0001
OHA ≥ 3	33,130 (34.14)	14,679 (35.03)	2118 (36.2)	2801 (35.46)	13,532 (32.7)	<0.0001

Abbreviations: <25/<25, the group with an initial BMI of <25 and <25 after 2 years; <25/≥25, the group with an initial BMI of <25 and ≥25 after 2 years; ≥25/<25, the group with an initial BMI of ≥25 and <25 after 2 years; ≥25/≥25, the group with an initial BMI of ≥25 and ≥25 after 2 years; eGFR; estimated glomerular filtration rate (mL/min/1.73 m^2^); BMI, body mass index; WC, waist circumference; SBP, systolic blood pressure; DBP, diastolic blood pressure; Tcholesterol, total cholesterol; HDL, high-density lipoprotein; α-glu inhibitor, α-glucosidase inhibitor OHA, oral hypoglycemic agents. * Alcohol consumption of ≥30 g/day.

**Table 2:** Baseline characteristics of patients according to body weight change.

Variables	Body Weight Change						
	Total (*n* = 97,029)	~−10% (*n* = 4930)	−10%~−5% (*n* = 13,804)	−5%~+5% (*n* = 66,493)	+5%~+10% (*n* = 8526)	+10%~ (*n* = 3276)	*p*-Value
Age, years	68.86 ± 9.11	72.28 ± 9.01	70.16 ± 8.78	68.31 ± 9.07	68.58 ± 9.17	70.04 ± 9.62	<0.0001
Sex, male	45,955 (47.36)	1566 (31.76)	5740 (41.58)	33,562 (50.47)	3855 (45.21)	1232 (37.61)	<0.0001
Smoking							<0.0001
Never	67,046 (69.10)	3904 (79.19)	10,062 (72.89)	44,587 (67.06)	6028 (70.7)	2465 (75.24)	
Former	18,625 (19.20)	577 (11.7)	2208 (16)	13,750 (20.68)	1603 (18.8)	487 (14.87)	
Current	11,358 (11.71)	449 (9.11)	1534 (11.11)	8156 (12.27)	895 (10.50)	324 (9.89)	
Alcohol consumption							<0.0001
none	75,960 (78.29)	4388 (89.01)	11,446 (82.92)	50,556 (76.03)	6781 (79.53)	2789 (85.13)	
mild	18,026 (18.58)	476 (9.66)	2045 (14.81)	13,608 (20.47)	1486 (17.43)	411 (12.55)	
heavy *	3043 (3.14)	66 (1.34)	313 (2.27)	2329 (3.05)	259 (3.04)	76 (2.32)	
Regular exercise	19,929 (20.54)	668 (13.55)	2567 (18.6)	14,647 (22.03)	1561 (18.31)	486 (14.84)	<0.0001
Hypertension	82,599 (85.13)	4216 (85.52)	11,680 (84.61)	56,461 (84.91)	7355 (86.27)	2887 (88.13)	<0.0001
Dyslipidemia	58,855 (60.66)	2790 (56.59)	8203 (59.42)	40,443 (60.82)	5382 (63.12)	2037 (62.18)	<0.0001
Income, <20%	16,657 (17.17)	899 (18.24)	2395 (17.35)	11,164 (16.79)	1559 (18.29)	640 (19.54)	<0.0001
eGFR	46.39 ± 12.72	45.47 ± 12.24	46.57 ± 12.11	46.59 ± 12.93	45.76 ± 12.34	44.67 ± 12.31	<0.0001
BMI, kg/m^2^	25.06 ± 3.30	22.45 ± 3.30	24.03 ± 3.10	25.28 ± 3.14	25.96 ± 3.38	26.35 ± 3.90	<0.0001
WC, cm	86.33 ± 8.67	82.16 ± 9.48	84.14 ± 8.51	86.75 ± 8.39	88.09 ± 8.73	88.52 ± 9.74	<0.0001
SBP, mmHg	130.7 ± 16.4	128.3 ± 17.4	129.4 ± 16.5	130.9 ± 16.2	132.2 ± 16.5	132.0 ± 17.4	<0.0001
DBP, mmHg	76.9 ± 10.3	75.8 ± 10.9	76.3 ± 10.4	77.0 ± 10.2	77.4 ± 10.4	77.4 ± 10.8	<0.0001
TC, mg/dL	180.9 ± 44.9	178.9 ± 43.2	179.6 ± 42.8	181.1 ± 45.2	181.6 ± 47.3	183.0 ± 43.3	<0.0001
Triglyceride, mg/dL	142.0 (141.5–142.4)	130.5 (128.7–132.3)	133.2 (132.1–134.3)	143.8 (143.3–144.4)	146.8 (145.2–148.4)	148.1 (145.6–150.6)	<0.0001
HDL, mg/dL	47.5 ± 14.0	48.4 ± 15.5	48.3 ± 15.5	47.4 ± 13.3	47.3 ± 14.9	47.1 ± 16.4	<0.0001
LDL, mg/dL	101.6 ± 45.2	101.7 ± 50.5	101.2 ± 38.3	101.6 ± 45.3	102.0 ± 52.6	102.8 ± 42.4	0.4008
Glu, mg/dL	131.1 ± 47.9	130.9 ± 60.4	129.7 ± 51.7	131.3 ± 46.0	130.9 ± 47.3	131.7 ± 50.4	0.0056
Insulin use	25,297 (26.07)	1598 (32.41)	3653 (26.46)	15,997 (24.06)	2823 (33.11)	1226 (37.42)	<0.0001
Anti-diabetes medication							
Sulfonylurea	61,710 (63.6)	3194 (64.79)	8772 (63.55)	42,121 (63.35)	5456 (63.99)	2167 (66.15)	0.0056
Metformin	67,768 (69.84)	3629 (73.61)	10,003 (72.46)	46,025 (69.22)	5861 (68.74)	2250 (68.68)	<0.0001
Meglitinides	5133 (5.29)	313 (6.35)	716 (5.19)	3261 (4.9)	577 (6.77)	266 (8.12)	<0.0001
TZD	9978 (10.28)	537 (10.89)	1361 (9.86)	6254 (9.41)	1299 (15.24)	527 (16.09)	<0.0001
DPP4 inhibitor	24,295 (25.04)	1340 (27.18)	3701 (26.81)	16,210 (24.38)	2234 (26.2)	810 (24.73)	<0.0001
α-glu inhibitor	22,237 (22.92)	1243 (25.21)	3198 (23.17)	14,775 (22.22)	2140 (25.1)	881 (26.89)	<0.0001
OHA ≥ 3	33,130 (34.14)	1903 (38.6)	4920 (35.64)	21,844 (32.85)	3176 (37.25)	1287 (39.29)	<0.0001

Abbreviations: eGFR; estimated glomerular filtration rate (mL/min/1.73 m^2^); BMI, body mass index; WC, waist circumference; SBP, systolic blood pressure; DBP, diastolic blood pressure; TC, total cholesterol; HDL, high-density lipoprotein; Glu, fasting glucose; TZD, Thiazolidine; α-glu inhibitor, α-glucosidase inhibitor OHA, oral hypoglycemic agents. * Alcohol consumption ≥30 g/day.

**Table 3:** Sensitivity analysis of incidence rates and hazard ratios of ESRD according to the weight change status after excluding ESRD with 4 years of follow-up.

Weight Changes	Number	ESRD	Follow-Up Duration, Person-Years	Incidence Rate, Per 1000 Person-Years	Model 1, HR (95% CI)	Model 2, HR (95% CI)	Model 3, HR (95% CI)
During (4 Years)							
≥−10%	5220	330	19,324.14	17.0771	1.69(1.50–1.90)	1.72(1.53–1.93)	1.76(1.56–1.98)
–10%~−5%	11,791	631	45,693.99	13.8093	1.20(1.10–1.31)	1.24(1.13–1.36)	1.26(1.15–1.37)
−5%~5%	40,319	2035	157,735.42	12.9014	1 (ref.)	1 (ref.)	1 (ref.)
5%~10%	5958	353	22,472.13	15.7083	1.26(1.12–1.41)	1.03(0.92–1.16)	1.01(0.91–1.14)
≥10%	2659	221	9633.21	22.9415	1.98(1.72–2.28)	1.42 (1.24–1.64)	1.34(1.16–1.54)

Abbreviations: ESRD, end-stage renal disease; BP, blood pressure; CI, confidential interval; ESRD, end-stage renal disease; HR, hazard ratio. Model 1, adjusted for age and sex; model 2, adjusted for age, sex, smoking, alcohol consumption, regular exercise, low-income status, use of insulin, number of oral hypoglycemic agents, diabetes duration, and previous histories of hypertension and dyslipidemia.; model 3, adjusted for age, sex, smoking, alcohol consumption, regular exercise, low-income status, use of insulin, number of oral hypoglycemic agents, duration of diabetes, previous histories of hypertension and dyslipidemia, and baseline body weight.

## Data Availability

Anonymized data are publicly available from the National Health Insurance Sharing Service and can be accessed at https://nhiss.nhis.or.kr/bd/ab/bdaba000eng.do, (accessed on 17 December 2021).

## Supplementary Materials

The following supporting information can be downloaded at: https://www.mdpi.com/article/10.3390/nu14010154/s1, Table S1: Incidence rates and hazard ratios of ESRD according to the baseline BMI and waist circumference; Table S2: Incidence rates and hazard ratios of ESRD according to the BMI and weight changes.
