# Supplementary material for: Underweight and Weight Change Increases End-Stage Renal Disease Risk in Patients with Diabetes: A Nationwide Population-Based Cohort Study
Source: Nutrients. 2021 Dec 29;14(1):154. doi: 10.3390/nu14010154 (PMC8747041; doi:10.3390/nu14010154)
Supplement: Supplementary file 1 [file nutrients-14-00154-s001.zip › nutrients-1487016-supplementary.pdf]

Supplementary Table S1. Incidence rates and hazard ratios of ESRD according to the baseline BMI and waist circumference.

Range	Number	ESRD	Follow-up Duration, Person-years	Incidence Rate, Per 1,000 person-years	Model 1, HR (95% CI)	Model 2, HR (95% CI)
Body Mass Index						
- 18.5	1330	116	5825.83	19.9113	1.27 (1.053, 1.531)	1.324 (1.098, 1.597)
18.5 - 23	23919	2173	125849.3	17.2667	1 (ref.)	1 (ref.)
23 - 25	24550	1987	135259.13	14.6903	0.812 (0.764, 0.863)	0.805 (0.758, 0.856)
25 - 30	40161	3087	223885.12	13.7883	0.761 (0.721, 0.804)	0.734 (0.694, 0.776)
30 -	7069	569	39094.87	14.5543	0.85 (0.775, 0.933)	0.758 (0.69, 0.832)
Waist circumference (Male/Female)						
- 80 / - 75	11624	1149	60376.74	19.0305	1.293 (1.201, 1.391)	1.373 (1.275, 1.478)
- 85 / - 80	17257	1422	94677.84	15.0194	0.997 (0.93, 1.068)	1.075 (1.004, 1.152)
- 90 / - 85	23985	1876	132652.16	14.1422	1 (ref.)	1 (ref.)
- 95 / - 90	26057	2142	142721.19	15.0083	1.031 (0.969, 1.098)	0.97 (0.912, 1.033)
- 100 / - 95	10078	715	55751.29	12.8248	1.174 (1.075, 1.281)	1.039 (0.952, 1.134)
100 - / 95 -	8028	628	43735.03	14.3592	1.408 (1.283, 1.544)	1.167 (1.064, 1.280)

Abbreviations: ESRD, end-stage renal disease; BP, blood pressure; CI, confidential interval; ESRD, end-stage renal disease; HR, hazard ratio. Model 1, adjusted for age and sex; Model 2, adjusted for model 1 variables as well as smoking, alcohol consumption, regular exercise, low-income status, use of insulin, number of oral hypoglycemic agents, diabetes duration, and previous histories of hypertension and dyslipidemia.

Supplementary Table S2. Incidence rates and hazard ratios of ESRD according to the BMI and weight changes.

Weight changes	Number	ESRD	Follow-up Duration, Person-years	Incidence Rate, Per 1,000 person-years	Model 1, HR (95% CI)	Model 2, HR (95% CI)	Model 3, HR (95% CI)	Cancer censoring HR (95% CI)
Body mass index changes								
< 25 / < 25	41901	3659	224174.76	16.3221	1 (ref.)	1 (ref.)		1 (ref.)
< 25 / ≥ 25	5851	530	31899.5	16.6147	1.04(0.95-1.14)	0.91(0.83-0.97)		0.91(0.82-0.99)
≥ 25 / < 25	7898	617	42759.5	14.4295	0.94(0.86-1.02)	0.95(0.87-1.04)		0.95(0.87-1.04)
≥ 25 / ≥ 25	41379	3126	231080.49	13.5278	0.82(0.78- 0.86)	0.80(0.76-0.84)		0.79(0.75-0.83)
Weight changes								
≥ –10%	4930	446	24027.42	18.5621	1.74(1.58-1.92)	1.61(1.46-1.77)	1.64(1.49-1.81)	1.64(1.48-1.81)
–10% ~ –5%	13804	1112	74174.51	14.9917	1.23(1.15-1.31)	1.21(1.13-1.29)	1.23(1.15-1.31)	1.23(1.15-1.31)
–5% ~ 5%	66493	5165	369388.01	13.9826	1 (ref.)	1 (ref.)	1 (ref.)	1 (ref.)
5% ~ 10%	8526	851	45708.04	18.6182	1.40(1.30-1.50)	1.17(1.09-1.26)	1.13(1.05-1.21)	1.13(1.05-1.22)
≥ 10%	3276	358	16616.28	21.5451	1.79(1.61-2.00)	1.35(1.21- 1.50)	1.25(1.12-1.39)	1.26(1.13-1.41)

Abbreviations: ESRD, end-stage renal disease; BP, blood pressure; CI, confidential interval; ESRD, end-stage renal disease; HR, hazard ratio. Model 1, adjusted for age and sex; Model 2 adjusted for model 1 variables as well as smoking, alcohol consumption, regular exercise, low-income status, use of insulin, number of oral hypoglycemic agents, diabetes duration, and previous histories of hypertension and dyslipidemia.; Model 3, adjusted for model 2 variables as well as 1st time body weight.
